# Resolution of radiation necrosis with bevacizumab following radiation therapy for primary CNS lymphoma

**DOI:** 10.18632/oncotarget.28222

**Published:** 2022-03-29

**Authors:** Eugene J. Vaios, Kristen A. Batich, Anne F. Buckley, Anastasie Dunn-Pirio, Mallika P. Patel, John P. Kirkpatrick, Ranjit Goudar, Katherine B. Peters

**Affiliations:** ^1^Department of Radiation Oncology, Duke University Medical Center, Durham, NC, USA; ^2^Department of Medicine, Division of Medical Oncology, Duke University Medical Center, Durham, NC, USA; ^3^Department of Pathology, Duke University Medical Center, Durham, NC, USA; ^4^Department of Neurology, UC San Diego Health, San Diego, CA, USA; ^5^Department of Pharmacy, Duke University Medical Center, Durham, NC, USA; ^6^Virginia Oncology Associates, Norfolk, VA, USA; ^7^Department of Neurosurgery, Duke University Medical Center, Durham, NC, USA; ^8^Department of Neurology, Duke University Medical Center, Durham, NC, USA

**Keywords:** radiation necrosis, brain tumor, primary CNS lymphoma, stereotactic radiosurgery, bevacizumab

## Abstract

Importance: Radiation necrosis (RN) is a rare but serious adverse effect following treatment with radiation therapy. No standard of care exists for the management of RN, and efforts to prevent and treat RN are limited by a lack of insight into the pathomechanics and molecular drivers of RN. This case series describes the outcomes of treatment with bevacizumab (BV) in two primary CNS lymphoma (PCNSL) patients who developed symptomatic biopsy-proven RN after whole brain radiation (WBRT) with a stereotactic radiosurgery (SRS) boost.

Observations: Patient 1 is a 52 year-old female with PCNSL treated with WBRT followed by an SRS boost. She developed symptomatic biopsy-proven RN, and initial treatment with tocopherol and pentoxifylline was unsuccessful. A 100% clinical and radiographic response was achieved with 4 cycles of BV. Patient 2, a 48 year-old male with PCNSL, presented with seizures and biopsy-proven RN after radiation therapy. Initial empiric treatment with tocopherol and pentoxifylline was unsuccessful. A 100% clinical and radiographic response was achieved with 3 cycles of BV.

Conclusions and Relevance: Monitoring for RN in patients with PCNSL treated with radiation therapy is warranted. BV is an efficacious treatment and a viable alternative to corticosteroids or surgical intervention.

## INTRODUCTION

Primary CNS lymphoma (PCSNL) is a rare and highly aggressive Non-Hodgkin’s lymphoma accounting for less than 3% of all primary CNS tumors [1]. Stereotactic radiosurgery (SRS) combined with reduced-dose whole brain radiation (rd-WBRT) has emerged as a promising method to improve local control following induction chemotherapy or in the setting of recurrent PCNSL [2]. With this regimen, rd-WBRT is delivered to a total dose of 23.4 Gy in 1.8 Gy daily fractions, followed by an SRS boost. While SRS is generally well-tolerated, radiation necrosis (RN) is a known complication that can develop months to years following radiation therapy [3].

The pathophysiology of RN is poorly understood, and no standard of care protocol exists to guide management. Corticosteroids play a key role in symptom management, but are limited by their deleterious long-term side effects [4]. The anti-VEGF-A monoclonal antibody bevacizumab (BV), has emerged as a promising non-invasive treatment modality to reverse neurological symptoms and radiographic changes resulting from RN while minimizing dependency on corticosteroids [5–9]. Here, we report the outcomes of two patients with PCNSL who developed symptomatic biopsy-proven RN that subsequently resolved with administration of a short course of BV. Both patients had no other underlying co-morbidities, including diabetes or cardiovascular disease.

## REPORT OF CASES

### Case 1

In April 2014, a 52-year-old female patient presented to her PCP with several weeks of confusion, decreased acuity of peripheral vision, vertigo, and emotional lability. Several weeks later, she presented to a local hospital with nausea, vomiting, and a generalized seizure. MRI brain revealed a 3.1 × 2.5 × 3.7 cm homogeneously enhancing mass with associated edema in the right frontal region. MRI spine was unremarkable. She underwent a biopsy, which confirmed PCNSL with a high KI-67 index (CD20+, PAX5+).

**Figure 1:**
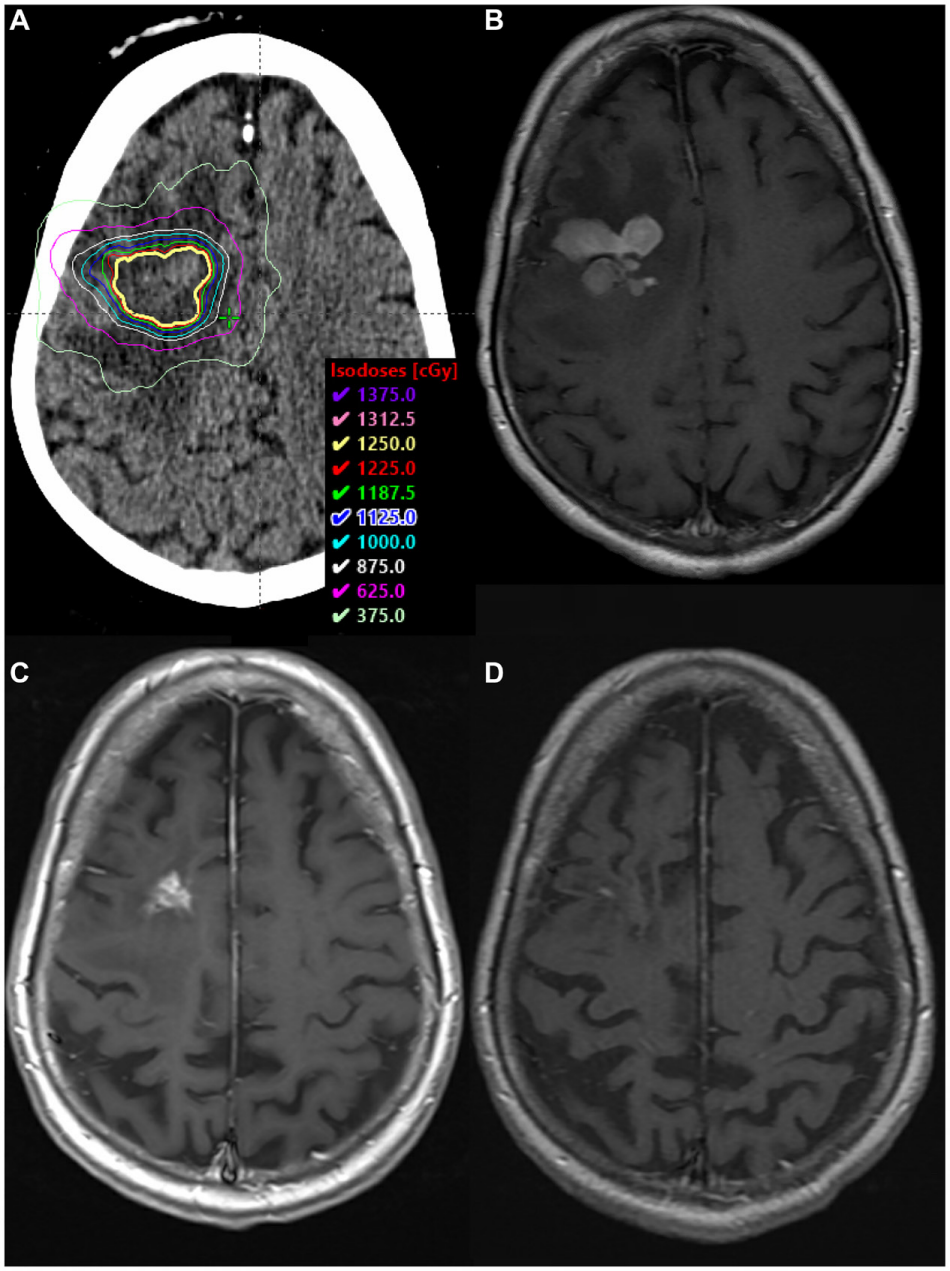
SRS boost treatment plan and MRI scans. (**A**) SRS boost treatment plan. (**B**) Enhancing residual disease prior to SRS boost on T1 sequence with contrast. (**C**) Biopsy-proven RN along the margin of the anterior body of the right lateral ventricle, within the SRS boost volume, on T1 sequence with contrast. (**D**) MRI >4 years after BV, demonstrating complete radiographic response on T1 sequence with contrast.

The patient was initiated on methotrexate (3.5 gm/m^2^) every 2 weeks for 6 cycles with concurrent weekly rituximab (RTX) (375 mg/m^2^) for 4 cycles, then every 2 weeks for 2 additional cycles. She received leucovorin rescue (20 mg) IV beginning 24 hours after each methotrexate infusion and was kept on dexamethasone 2 mg daily. She remained on levetiracetam 500 mg orally twice daily for seizure prophylaxis and pentamidine monthly for PCP prophylaxis. Levetiracetam was increased to 1000 mg twice a day following a breakthrough seizure prior to cycle 4 of RTX. Repeat imaging after her 5th cycle of methotrexate revealed a 3.7 × 2.7 × 4.3 cm lesion with increased surrounding T2 signal abnormality within the right frontoparietal lobes and new extension into the corpus callosum, concerning for disease progression. Per our institution’s practice, she was treated with rd-WBRT followed by an SRS boost of 12.5 Gy to the residual enhancing volume present on MRI 1 week before the boost treatment (Figure 1A and 1B). She had a complete radiographic response and no recurrent seizures.

**Figure 2:**
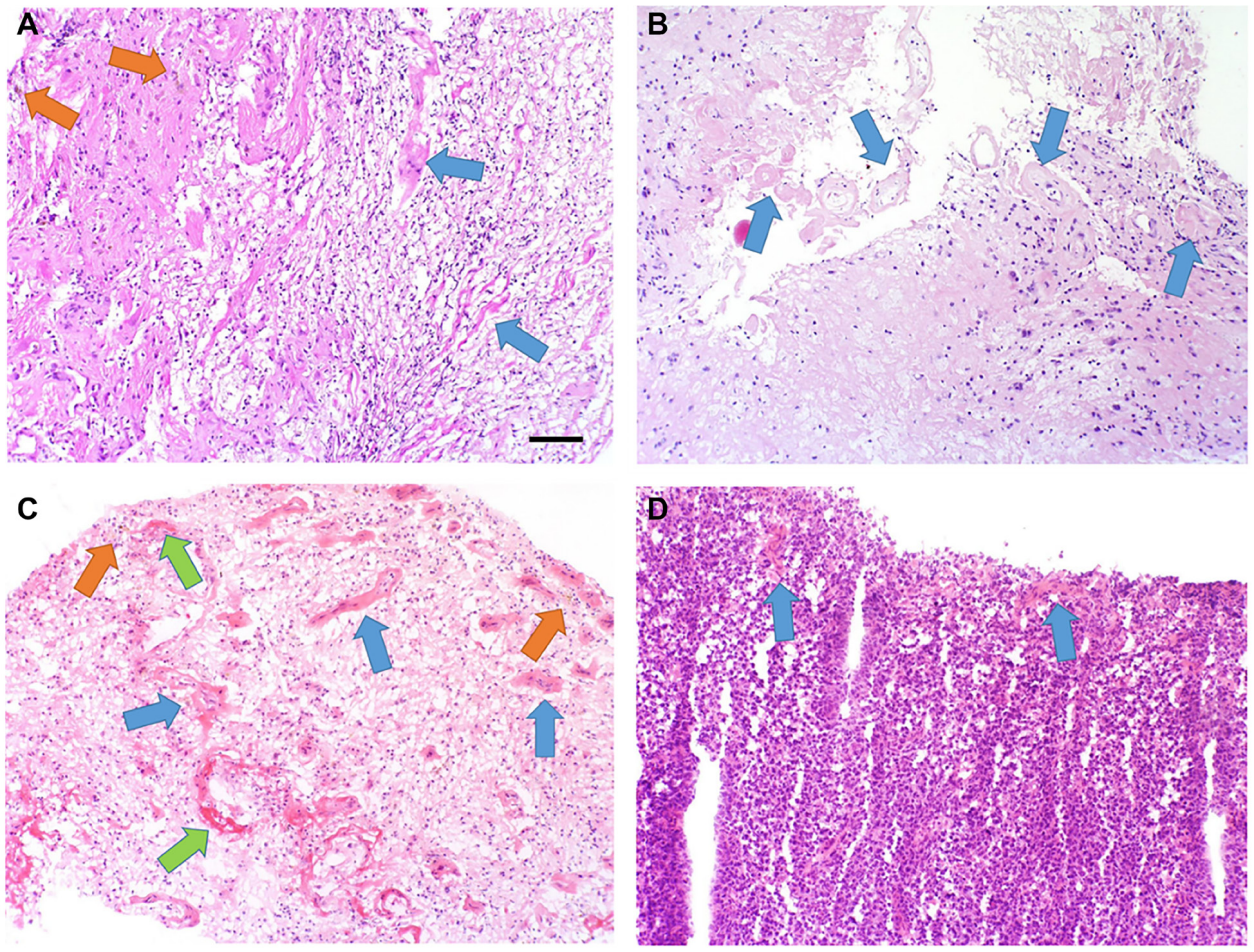
Pathology results. (**A**) H&E-stained tissue sections of brain biopsy show areas of parenchymal gliosis with reactive astrocytes (upper left area) and tissue necrosis with macrophages and other inflammatory cells (lower right area). Foci of hemosiderin (orange arrows) indicate prior vascular leakage and microhemorrhage, and hyalinized and disrupted blood vessels are evident (blue arrows). (**B**) H&E-stained tissue sections of brain biopsy show more hypocellular areas (lower left) with loss of neurons and glia, with fewer macrophages and inflammatory cells. Hyalinized and disrupted blood vessels are prominent and frequent. (**C**) H&E-stained tissue sections of brain biopsy show diffuse parenchymal edema with gliosis and inflammation. Foci of hemosiderin (orange arrows) indicate prior vascular leakage and microhemorrhage, and hyalinized and disrupted blood vessels are evident (blue arrows). Foci of vascular fibrinoid necrosis are noted (green arrows). (**D**) H&E-stained tissue sections of brain biopsy show diffuse parenchymal involvement by large neoplastic B-cells. A few hyalinized blood vessels are present, indicating some radiation treatment effect (blue arrows). 10× objective, scale bar = 100 micrometers.

Fourteen months after completion of radiation therapy, surveillance MRI revealed a new enhancing nodule within the radiation boost volume. The lesion was located along the margin of the anterior body of the right lateral ventricle near the biopsy site, measuring 4 × 8 × 12 mm. Repeat imaging 3 months later revealed interval progression, with more prominent surrounding vasogenic edema. Symptoms included increased word-finding difficulty. Stereotactic biopsy confirmed RN (Figure 2A), and she was started on pentoxifylline 400 mg three times daily and tocopherol 1000 units daily.

The nodular enhancement/FLAIR signal in the right periventricular white matter remained stable for thirteen months, until surveillance imaging revealed a slight interval increase in enhancement (Figure 1C). Symptoms included new left lower extremity weakness and dizziness. PET/CT confirmed hypometabolic activity within the concerning lesion, and repeat biopsy revealed RN (Figure 2B). She received BV 7.5 mg/kg every three weeks for four total doses. She ultimately achieved a complete radiographic response, and symptoms resolved without necessitating steroids. She has had no evidence of progression for over four years (Figure 1D).

### Case 2

A 48-year-old man was diagnosed with PCNSL in November 2016 after experiencing a year of unresolved vision changes. Left vitrectomy revealed monoclonal B cells consistent with B-cell lymphoma. MRI demonstrated two nodular contrast-enhancing T2 FLAIR hyperintense lesions: an 8 × 5 mm lesion in the left anterior insula and a 7 × 6 mm lesion in the left parietal region. Biopsy of the left anterior insula revealed high-grade, diffuse large B-cell lymphoma (DLBCL).

The patient was treated with methotrexate, rituximab, and temozolomide (MTR) over a 10-week period based on a protocol from Glass et al. with modifications [10]. Methotrexate and rituximab (MTX-R) were given every 2 weeks for a total of 5 cycles. Temozolomide (TMZ) was given on weeks 4 and 8 at 100 mg/m^2^ for 5 days. MRI following 5 cycles of MTR revealed a moderate increase in the left frontal lesion with near resolution of the left parietal lesion, consistent with a partial response. The patient then completed two additional cycles of MTX-R and one five-day cycle of TMZ.

**Figure 3:**
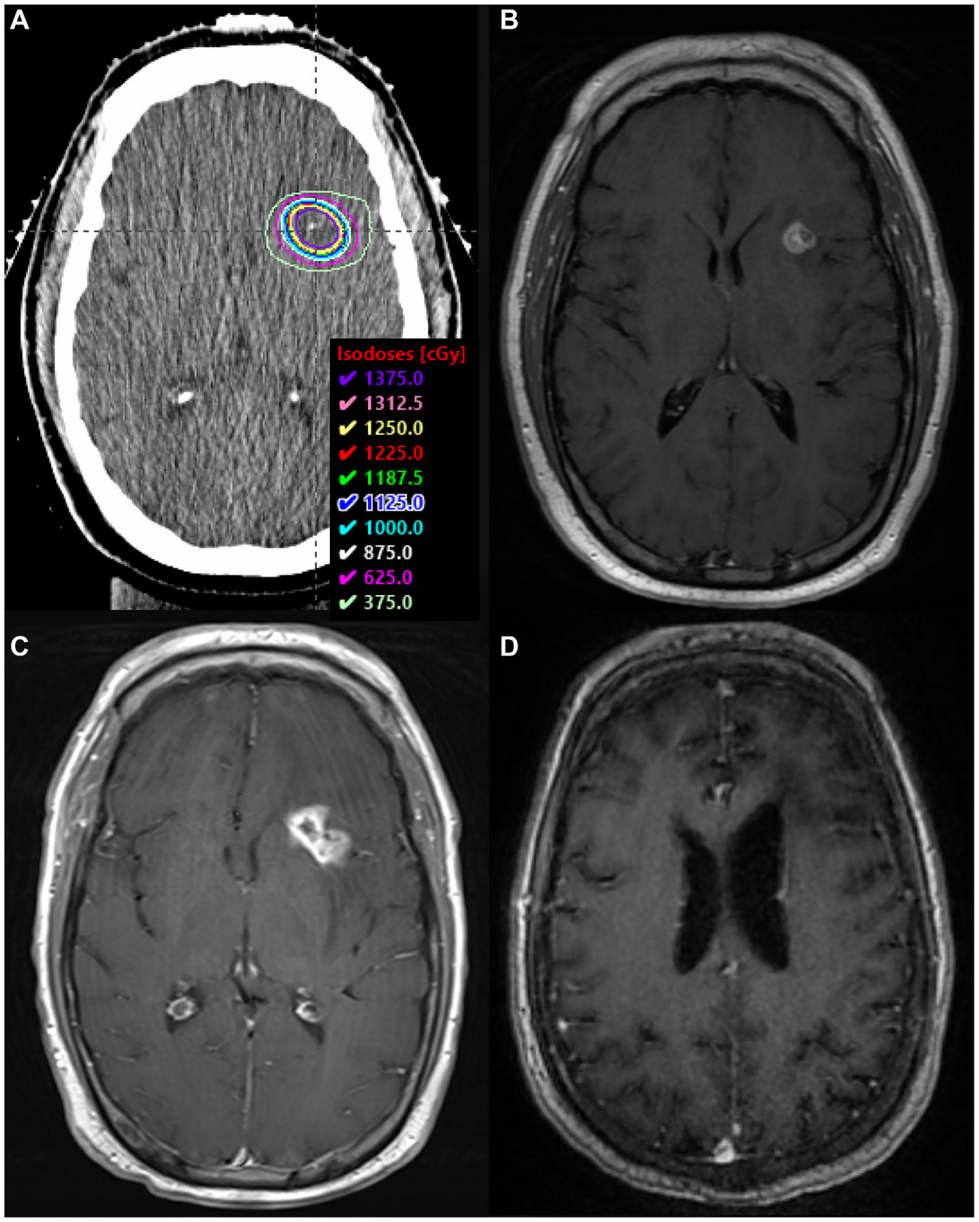
SRS boost treatment plan and MRI scans. (**A**) SRS boost treatment plan. (**B**) Enhancing residual disease prior to SRS boost on T1 FLAIR sequence with contrast. (**C**) Biopsy-proven RN along the left internal capsule, within the SRS boost volume, on T1 sequence with contrast. (**D**) MRI >2.5 years after BV, demonstrating complete radiographic response on T1 sequence with contrast.

At the conclusion of treatment, MRI again revealed partial response with no reduction in the left frontal lesion. He received rd-WBRT with a 12.5 Gy SRS boost to the residual lesion in the left frontal lobe (Figure 3A and 3B). He then completed 10 cycles of consolidative TMZ 150 mg/m^2^ for 5 days in 28-day cycles. Intravitreal injections of MTX-R were added six months into the treatment.

Four months after initiating his intravitreal injections, MRI revealed a new enhancing nodule in the left anterior basal ganglia. This was presumed to be RN, and he was empirically started on pentoxifylline 400 mg three times daily and tocopherol 1000 units daily. Three months later, he presented with generalized seizures and MRI demonstrated a new 14-mm enhancing lesion along the left pre-central gyrus. Additionally, there was interval increase in the size of the previously seen left internal capsule lesion, with new central cavitation, increased T2 FLAIR hyperintensity suggesting vasogenic edema, and a 4-mm left-to-right midline shift (Figure 3C). He was started on high-dose steroids and levetiracetam. Biopsy revealed RN in the left internal capsule (Figure 2C) and recurrent DLBCL (CD45 99%, MGMT positive 99%) in the left motor strip (Figure 2D).

In the setting of recurrent disease, he completed another three months of MTX-R for 5 cycles followed by TMZ consolidation for 10 cycles. Given his biopsy-proven RN, he also received BV 7.5 mg/kg IV every 3 weeks for three total doses. BV was initiated after the fourth cycle of MTX-R. After two cycles of BV, MRI demonstrated a decrease in size and T2 hyperintensity at the location of his biopsy-proven RN. Both lesions continued to resolve on subsequent imaging, with stable T2 hyperintensity and complete loss of contrast enhancement (Figure 3D). Over two years later, he remains clinically and radiographically stable without seizures.

## DISCUSSION

To our knowledge, this is the first report to highlight outcomes following the administration of BV in patients with PCNSL who developed symptomatic biopsy-proven RN after radiation therapy. Data on RN largely derives from studies of patients with brain metastases. Proposed risk factors include patient age, cardiovascular comorbidities, tumor histology, radiation technique (e.g., stereotactic radiosurgery), radiation modality (proton versus photon), radiation dose, volume treated, fractionation, and use of concurrent and/or adjuvant systemic therapy [3, 11]. While SRS is generally well-tolerated, the complication of cerebral RN can develop months to years following radiation therapy. RN presents an ongoing diagnostic challenge as it mimics recurrent tumor radiographically, and aside from surgical biopsy, we lack effective clinical tools to distinguish RN from recurrence [12–16]. Additionally, the clinical course of patients with RN is variable and can be associated with significant morbidity. Cognitive decline, neurologic deficits, seizures, and signs of increased intracranial pressure are reported in more than 60% of patients with RN [13, 17, 18]. Symptoms may resolve spontaneously for patients, but can progress in others, necessitating medical and surgical interventions.

No standard of care exists for the management of RN, and efforts to prevent and treat RN are limited by a lack of insight into the pathomechanics and molecular drivers of RN. Corticosteroids are frequently used to reduce vasogenic edema and the associated mass effect, thought to result from radiation-induced disruption to the blood-brain barrier and release of inflammatory cytokines. However, use of corticosteroids is limited by their deleterious side effects. Other non-invasive treatments include hyperbaric oxygen, Pentoxifylline, vitamin E, and nerve growth factor [19–22]. Surgical intervention is sometimes necessary for acutely symptomatic RN in cases with significant mass-effect. More recently, minimally invasive techniques, such as laser interstitial thermal therapy (LITT) have been explored as a solution for lesions that are surgically inaccessible, located in eloquent areas of the brain, or in settings where surgery is contraindicated [23].

BV, a VEGF-A monoclonal antibody, is a non-invasive alternative which has demonstrated efficacy in reversing radiographic changes and neurologic deficits in patients with RN. The efficacy of BV stems from the proposed mechanism by which radiation therapy induces RN. RN is thought to result from damage to vascular endothelial cells, triggering fibrinoid necrosis, endothelial cell proliferation, perivascular edema, and upregulation of hypoxia-inducible factor 1α (HIF1α). HIF1α, expressed by astrocytes and endothelial cells, promotes VEGF expression which in turn potently stimulates angiogenesis, vascular permeability, brain edema, and the resulting hypoxia and necrosis [24–26]. BV-mediated binding and downregulation of VEGF is therefore thought to mitigate cerebral RN by interrupting the downstream effects of tissue hypoxia.

The observed efficacy of BV for the treatment of RN derives mostly from studies of primary and metastatic brain tumors. In a small, randomized double-blind trial of patients with primary brain tumors and head-and-neck cancer, BV was associated with improvement in neurologic symptoms, decrease in steroid dependence within 3 months, improvement in learning and memory, and a radiographic reduction in enhancement and FLAIR signal abnormality [5]. BV was dosed at 7.5 mg/kg at 3-week intervals for 2 cycles, with the option of an additional 2 cycles in responders without adverse effects. In a systematic review that included 30 patients with high-grade gliomas treated with fractionated radiation therapy, Lubelski et al. reported improved neurologic symptoms in 70% and a partial or mixed response in 22% of patients [9]. All patients were able to reduce their dependance on dexamethasone, and BV was delivered as infusions of 5–10 mg/kg every 2 weeks over an average of 4–8 weeks. A recent meta-analysis of patients with brain metastases similarly reported improvement in symptoms and discontinuation of steroids in the majority of patients following administration of BV [27]. However, single patient case series have reported acquired drug resistance and paradoxical neurological worsening during BV treatment in patients with brain metastases and primary gliomas, respectively [28, 29]. Additionally, BV can be associated with systemic toxicity, including severe hypertension, sinus thrombosis, bowel perforation, pulmonary emboli, and wound dehiscence [30]. Given the financial cost of treatment with BV, ongoing investigations of its cost-effectiveness and clinical efficacy across tumor histologies are warranted [9].

This case series, to our knowledge, is the first to report the use of BV in PCNSL patients with symptomatic biopsy-proven RN. It has been noted that BV provides a benefit in the treatment of multiple different CNS neoplasms including gliomas, brain metastases, and mesenchymal neoplasms [5]. This study adds to these insights by demonstrating that BV can also benefit patients with CNS DLBCL. This is meaningful because each type of neoplasm can be assumed to have a different pathologic relationship with the blood vessels and brain parenchyma, and each has a different treatment regimen with implications for radiation sensitivity and response to BV. Future studies are needed to further characterize the incidence and risk factors for RN in in patients with PCNSL, as well as the optimal dose and duration of BV for the treatment of RN in this patient population.
