# Addendum: Resolution of radiation necrosis with bevacizumab following radiation therapy for primary CNS lymphoma

**DOI:** 10.18632/oncotarget.28292

**Published:** 2022-10-20

**Authors:** Eugene J. Vaios, Kristen A. Batich, Anne F. Buckley, Anastasie Dunn-Pirio, Mallika P. Patel, John P. Kirkpatrick, Ranjit Goudar, Katherine B. Peters

**Affiliations:** ^1^Department of Radiation Oncology, Duke University Medical Center, Durham, NC, USA; ^2^Department of Medicine, Division of Medical Oncology, Duke University Medical Center, Durham, NC, USA; ^3^Department of Pathology, Duke University Medical Center, Durham, NC, USA; ^4^Department of Neurology, UC San Diego Health, San Diego, CA, USA; ^5^Department of Pharmacy, Duke University Medical Center, Durham, NC, USA; ^6^Virginia Oncology Associates, Norfolk, VA, USA; ^7^Department of Neurosurgery, Duke University Medical Center, Durham, NC, USA; ^8^Department of Neurology, Duke University Medical Center, Durham, NC, USA

**Consent was added to this article:** Informed consent was obtained from each patient and the study was approved by our institution.

Original article: Oncotarget. 2022; 13:576–582. 576-582. https://doi.org/10.18632/oncotarget.28222

